# Associations between myeloperoxidase and paraoxonase-1 and type 2 diabetes in patients with ischemic heart disease

**DOI:** 10.1186/s12872-022-02928-8

**Published:** 2022-12-03

**Authors:** Katarzyna Nessler, Rafal Grzybczak, Michal Nessler, Jarosław Zalewski, Grzegorz Gajos, Adam Windak

**Affiliations:** 1 Department of Family Medicine, Chair of Internal Medicine and Gerontology, Jagiellonian University Medical College in Krakow, 4 Bochenska str, 31-061 Krakow, Poland; 2 Department of Cardiac Rehabilitation, Institute of Cardiology, Jagiellonian University Medical College, 80 Pradnicka str, 31-202 Krakow, Poland; 3 Burns and Plastic Surgery Centre of Malopolska, Rydygier Memorial Hospital, Os. Zlotej Jesieni 1, 31-826 Krakow, Poland; 4 Department of Coronary Disease and Heart Failure, Institute of Cardiology, Jagiellonian University Medical College, John Paul II Hospital, 80 Pradnicka str, 31-202 Krakow, Poland

**Keywords:** Dysfunctional high-density lipoprotein, Myeloperoxidase (MPO), Paraoxonase-1 (PON-1), Type 2 diabetes, Ischemic heart disease

## Abstract

**Background:**

The phrase “dysfunctional high-density lipoprotein” has been developed in the literature to describe the particle which loses its basic role- anti-oxidative and anti-inflammatory activity. In this porcess, the significance of enzymes- pro-oxidant myeloperoxidase (MPO) and antioxidant paraoxonase-1 (PON-1) from the perspective of HDL-C function has been noted.

**Aims:**

The objective of this study was to analyze the associations between two enzymes –MPO and PON-1 and type 2 diabetes (T2DM) in patients with ischemic heart disease (IHD).

**Methods:**

An observational cross-sectional study including 70 patients with IHD of whom 35 had also T2DM, and 35 had no T2DM. Laboratory tests (MPO, PON-1, fasting glucose, glycated hemoglobin, total cholesterol, triglycerides, high-density lipoprotein, low-density lipoprotein, and high-sensitivity C-reactive protein) were performed.

**Results:**

The study revealed a significant difference in the serum concentration of the enzymes between patients with IHD with and without T2DM. Our results showed increased MPO concentration levels in diabetic patients. The analysis also revealed that T2DM is independently associated with an increase in MPO levels. Simultaneously, a decrease in PON-1 levels was observed in patients with T2DM. The study also revealed that T2DM is independently associated with a decrease in PON-1 levels.

**Conclusions:**

In patients with type 2 diabetes the profile of enzymes involved in high-density lipoprotein metabolism in patients with IHD is worse than in patients without T2DM. The increase in the levels of MPO, an enzyme with oxidative and atherogenic properties and on a decrease in PON-1 levels, an enzyme with antioxidant and atheroprotective properties is observed.

## What’s new?

The presented study shows some of the possible directions for future research to bring us closer to the full understanding of the dysfunctional high-density lipoprotein and the role of myeloperoxidase (MPO) and paraoxonase-1 (PON-1) in patients with ischemic heart disease and type 2 diabetes.

The study shows that the profile of enzymes involved in high-density lipoprotein HDL-C metabolism in patients with ischemic heart disease and type 2 diabetes is significantly worse than in patients without dibetes. The disease acts as an independent factor associated with an increased concentration of myeloperoxidase, an enzyme with oxidative and atherogenic effects, and at the same time independently reduces the levels of paraoxonase-1, an enzyme with antioxidant and atheroprotective properties.

Our findings may have important prognostic significance; however, we are aware that they need to be confirmed in a longer follow-up of a larger group of patients. Our paper provides new insight in the discussed topic.

## Introduction

Oxidative stress and inflammation play the direct role in the initial and progression of atherosclerosis plaques and development of ischemic heart disease (IHD) [1]. A high-density lipoprotein (HDL) particle has a role of cardiovascular protection mainly by reverse cholesterol transport. Despite the atheroprotective properties of HDL particles, there are situations where despite normal or elevated levels of HDL a significant atherosclerotic change appears [2–4]. The phrase “dysfunctional HDL” has been developed in the literature to describe HDL particle which loses its basic role- anti-oxidative and anti-inflammatory activity [5–7].

The main protein of HDL particles with the antioxidative function is Apolipoprotein A-I (ApoA-I), the action of which is strictly dependent on the enzyme known as myeloperoxidase (MPO). According to some authors there is a connection between a high concentration level of MPO and the progression of development of atherosclerotic plaque [8].

Present in neutrophils and monocytes, MPO is an enzyme that catalyzes the formation of the highly oxidizing hypochlorous acid. The enzyme induces the expression of the endothelial cell tissue factor and a modification of ApoA-I that impairs its ability to promote cholesterol efflux [9]. The levels of plasma circulating MPO may be correlated with the degree of endothelial dysfunction. It was demonstrated that atherosclerotic plaque is considerably enriched with MPO [10].

The antioxidant activity of HDL particles is related to the concentration of paraoxonase-1 (PON-1). The enzyme is synthesized in the liver and makes up HDL particles that contain ApoA-I. PON-1 is known to slow down the oxidation of lipids and contribute to the antioxidant and atheroprotective activity of HDL particles. The enzyme owes its properties to the ability not only to eliminate potential oxidants but also to degrade their end products to non-toxic substances. The activity of PON-1 has recently been reported to decrease in step with falling HDL cholesterol levels in type 2 diabetes (T2DM) patients as compared to healthy subjects [11]. The authors of the study also observed that the progression of diabetes may be accompanied by a substantial decrease in PON-1 activity. Accordingly, it was suggested that, as a state of increased oxidative stress, diabetes may cause the antioxidant activity of the PON-1 enzyme to diminish [11].

Diabetes is the state of elevated oxidative stress. Studies conducted to date have revealed an increased production of the reactive forms of oxygen as the result of tissue damage in diabetic patients [12–14].

The objective of our study was to analyze the associations between type 2 diabetes and the concentration of two enzymes – myeloperoxidase and paraoxonase-1 in patients with ischemic heart disease.

## Methods

### Study population

Participants were recruited from the patients who were hospitalized in the Department of Coronary Heart Disease and Heart Failure at John Paul II Hospital in Krakow in the period of February 2015 to December 2017. The inclusion and exclusion criteria are shown in table 1.

**Table 1:** The study inclusion and exclusion criteria

inclusion criteria	exclusion criteria
• age > 18 years, stable IHD lasting for at least 2 years • intake of Atorvastatin 20 mg or Rosuvastatin 10 mg for at least 2 years.	• diabetes different than type 2, • acute coronary incident/intervention in last 3 months, • acute infection, • thyroid diseases, history of neoplasm, immunodeficiency disorders, • patients after surgical procedures in previous 3 months, • pregnancy, • intake of: insulin, statins of another type or dose than Atorvastatin 20 mg or Rosuvastatin 10 mg, non- steroid anti-inflammatory drugs (NSAID) and/or steroids, fibrates, fibrinolytics.

The study participants were recruited into the two groups with and without co-existing type 2 diabetes mellitus. Matched sampling recruitment was performed to eliminate number of confounding factors. The following matching criteria were applied: patients’ gender, age, smoking status, body mass index (BMI) and the kind and dosage of statins used in treatment.

Patients who met the inclusion and exclusion criteria and had co-existing type 2 diabetes non-treated by insulin injections were assigned to the diabetes group (DM). Patients without accompanying diabetes were qualified to the group without diabetes (nDM).

### Definitions

Diabetes was defined as fasting plasma glucose (FPG) ≥ 7.0 mmol/L (measured twice), hemoglobin A1c (HbA1c) ≥ 6.5% or diagnosis made earlier by a healthcare professional and noted in the patient’s medical records [15]. Hypertension was defined by systolic blood pressure ≥ 140 mmHg, diastolic blood pressure ≥ 90 mmHg, or self-reported previous diagnosis of hypertension made by a physician.

Weight and height were measured in light underwear on calibrated medical scale. Body mass index (BMI) was calculated as the weight in kilograms divided by height in meters squared. Waist circumference was measured using an anthropometric tape at a level on the skin midway between the mean point of iliac peak and the inferior border of the last rib at the level of the umbilicus. Hip circumference was measured over the widest part of the gluteal region at the level of pubic tubercle. Waist-to-hip ratio (WHR) was calculated by waist circumference (cm) divided by hip circumference (cm).

Current smoking was defined as having smoked at least 100 cigarettes in a lifetime and currently smoking cigarettes [16]. Pack-years (i.e., the number of cigarette packs smoked per day multiplied by the years of smoking) were calculated for all active smokers (i.e., those who currently smoke or have smoked in the past 10 years). To assess the level of physical activity, we used the SQUASH form (the Short Questionnaire to Assess Health enhancing physical activity) [17], drawn up based on the Ainsworth compendium that classifies various forms of physical activity in terms of their energy expenditure [18]. The final score, based on answers to the questionnaire and relevant conversion indices, was represented as a continuous variable, with a higher score indicating a higher level of physical activity.

The IHD was defined according to 2006 ESC guidelines as reported in the discharge card diagnosis including coronary heart disease, myocardial infarction, or stroke.

### Measurements

A standard questionnaire was administered by the Primary Investigator (PI) of the study to obtain information on sociodemographic characteristics, personal and family medical history, and lifestyle risk factors of all participants. Clinical examinations, including weight, height, and blood pressure, were conducted by a trained staff group according to a standard guideline. Serum samples were obtained between 6:00 a.m. and 9:00 a.m. after fasting for at least 8 h. Venipuncture was performed in the median cubital vein, and centrifugation and dispensing were completed within 1 h. All samples were cold-chained, stored and transported to a central medical laboratory for testing within 2–4 h.

### Laboratory methods

HbA1c was assessed by high-performance liquid chromatography (HPLC). Plasma glucose and lipid profiles including total cholesterol (TC), triglycerides (TG), high-density lipoprotein (HDL) and low-density lipoprotein (LDL) were measured by enzymatic chromatography. High-sensitivity C-reactive protein (hsCRP) was measured with the use of immunoturbidimetric assay.

The separate samples were collected into a disposable EDTA vacuum tube and immediately centrifuged at 1000×g for 10-15 minutes at the temperature of 4-8 °C. Once duly secured, they were placed in Eppendorf tubes and stored at − 80 °C, for the further analysis of MPO and PON-1 concentrations.

Serum enzyme levels were tested manually by the direct ELISA method (enzyme-linked immunosorbent assay). To estimate the concentration of MPO, the study employed platelet-poor EDTA plasma along with the Human Myeloperoxidase Quantikine ELISA Kit DMYE00B with the anti-MPO polyclonal antibody. For the PON-1 measurement, it used EDTA plasma and the Human Serum Paraoxonase-1 (PON-1) ELISA kit Elabscience with the anti-PON-1 monoclonal antibody.

### Statistical analysis

Study results were fed into the database through the Microsoft Access (Microsoft Inc.) software, and, to verify that they were introduced correctly, a random sample of records (10%) were double-checked. The statistical analysis of the data was performed with the Statistica 12 (Statsoft Inc.) package, adopting the universally accepted statistical significance level of α = 0.05. To represent patient characteristics (i.e., the group as a whole and the two subgroups: DM and nDM), the analysis used frequency distributions for nominal and ordinal traits, the mean with standard deviation, the median with interquartile ranges, as well as minimum and maximum values for interval (quantitative) variables.

The DM and nDM groups were compared with the chi-square test for categorical variables and the t-Student test was used for a normally distributed quantitative variable (age). To investigate the correlations between different pairs of variables, depending on data distribution, the study calculated either the Pearson correlation coefficient or the Spearman rank correlation coefficient. The analysis studied the correlation between MPO and PON-1 enzymes and variables such as sex, age, lifestyle, chronic conditions, anthropometric measurements, physical exam results, and the basic parameters of carbohydrate and lipid metabolism. Based on multiple regression analysis, the variables that independently influence MPO and PON-1 levels were established. Due to the small size of the DM and nDM groups, multiple regression analysis models could only be constructed for the entire patient group, with diabetes introduced as an independent variable.

### Ethics

The study received full approval by the Jagiellonian University Bioethics Committee, number KBET/25/B/2013. All qualified patients were fully informed about the study protocol and rules and the written informed consent was obtained from those who qualified. All procedures adhered to the ethical standards of the responsible committee on human experimentation (institutional and national) and the Helsinki Declaration of 1975, as revised in 2008.

## Results

### Characteristics of participants

The study included 75 patients. Owing to the hemolysis of the biological material, 5 samples could not be used. In the end, data from 70 patients served as the basis for calculation. Most patients in the study were men (*n* = 54, 77.1%), accounting for 27 patients in each group. The mean (SD) age was 61.3 (5.8), min. 45, max. 73. There was no significant difference between groups regarding the patients’ age, with mean age 61.5 (5.8) in the DM and 61.1(6.0) in the nDM group.

Most subjects were married and lived in urban areas. Out of DM group, 7 patients were taking sulfonylurea derivatives (20%), and 14 were on Metformin (40%). Alpha-glucosidase inhibitor was taken by 1 patient (2,86%).

In accordance with the study design, patients in the DM group did not differ significantly from those in the nDM group in terms of age, gender, BMI, smoking status or the type and dosage of statins used in treatment.

Other patients’ demographics, along with their division into groups, are detailed in table 2. Lipid parameters among participants with (DM) and without DM2 (nDM) were presented in table 3.

**Table 2:** Categorical patients’ characteristics among participants with (DM) and without DM2 (nDM)

Feature					P
		All patients N 70	DM N 35	nDM N 35	
Gender	N (%)				1.0
	Women	16	8	8	
	Men	54	27	27	
Marital status	N (%)				0.76
	Single	2	1	1	
	Married	60	29	31	
	Divorced	4	3	1	
	Widowed	4	2	2	
Place of living	N (%)				0.19
	Village	16	10	6	
	City < 100,000	14	8	6	
	City > 100,000	40	17	23	
Education	N (%)				0.12
	Less than high school	26	15	11	
	High school equivalent	31	17	14	
	University education	13	3	10	

*DM* Participants with diabetes type 2, *nDM* Participants without diabetes type 2,

**Table 3:** BMI, glucose, HbA1c, lipid parameters, hsCRP [mg/l] among participants with (DM) and without DM2 (nDM)

Feature				P
	All patients *N =* 70 (SD)	DM *N =* 35 (SD)	nDM *N =* 35(SD)	
BMI	31,37 ± 4,2	33,87 ± 4,4	29,04 ± 3,3	0,202
TC [mmol/l]	4,38 ± 1,19	4,06 ± 0,86	4,70 ± 1,38	0,022
LDL-C [mmol/l]	2,29 ± 0,90	2,03 ± 0,74	2,54 ± 0,98	0,020
TG [mmol/l]	1,81 ± 1,36	1,80 ± 1,16	1,82 ± 1,55	0,951
HDL-C [mmol/l]	1,26 ± 0,40	1,22 ± 0,44	1,31 ± 0,34	0,363
hsCRP [mg/l]	1,55 ± 1,08	1,60 ± 1,23	1,50 ± 0,92	0,684
Glucose [mmol/l]	6,19 ± 1,82	7,05 ± 2,26	5,32 ± 0,28	0,000
HbA1c [%]	6,04 ± 0,87	6,49 ± 1,01	5,59 ± 0,28	0,000

*DM* Participants with diabetes type 2, *nDM* Participants without diabetes type 2, *HbA1c*-Hemoglobin A1c, *SD* Standard deviation

### Diabetes and the studied enzymes

**Fig. 1:**
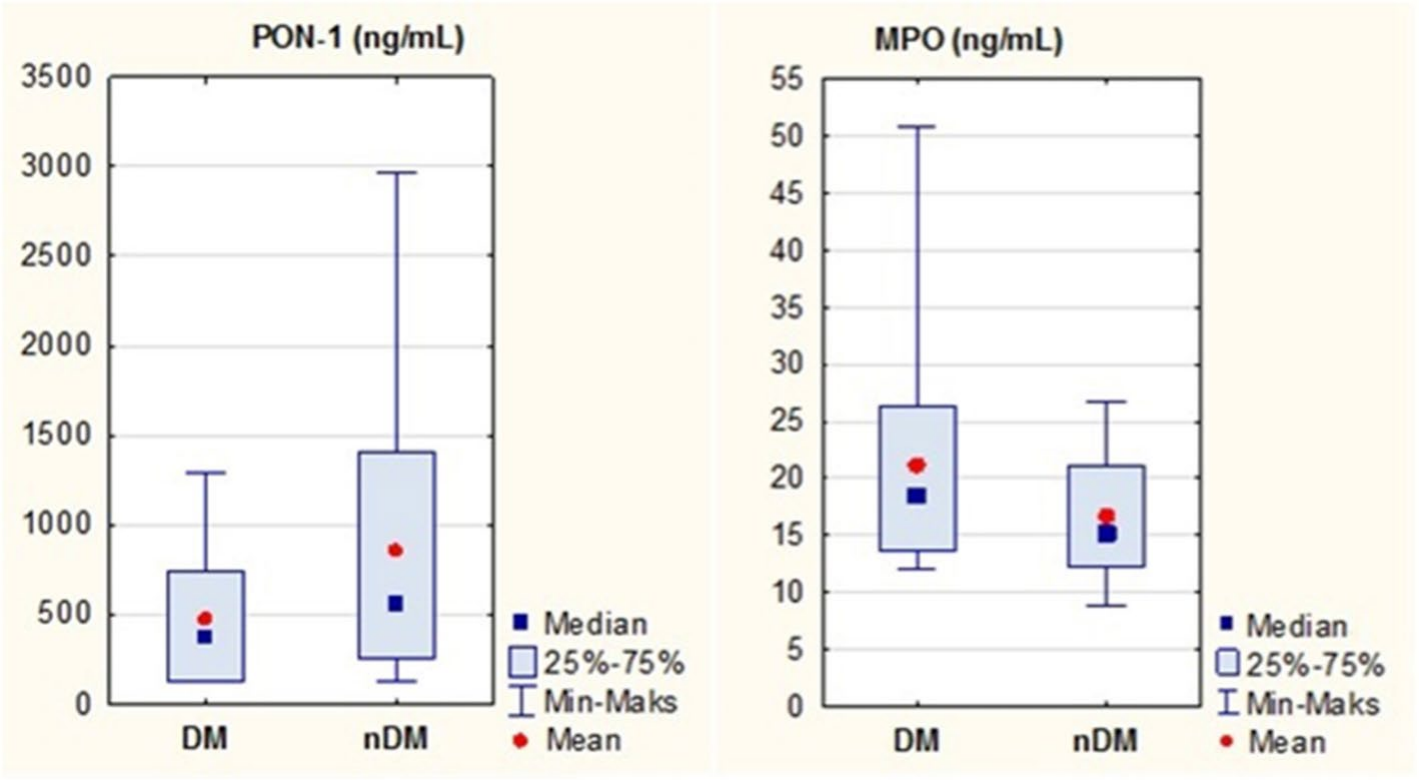
MPO and PON-1 levels in the study group

Statistically significant differences in the levels of both enzymes were observed between the study groups, as shown in fig. 1.

Patients with diabetes showed significantly higher MPO concentration levels in serum, *P =* 0.02.

The concentration of PON-1, on the other hand, was significantly higher in non-diabetic subjects, *P =* 0.04.

### MPO

MPO levels also increased as a function of age in patients without diabetes, R = 0.44. Other correlations with physical activity, dietary habits, substance use, anthropometric measurements, and physical exam results were not shown to be statistically significant.

No significant correlations were observed between the concentration of MPO and that of inflammatory indicators or carbohydrate and lipid metabolism parameters.

Mean MPO levels were significantly elevated in non-diabetic patients with heart failure. Diabetic patients who have not suffered a heart attack also had significantly higher MPO levels as compared to those without a heart attack in the interview, as shown in table 4.

**Table 4:** MPO levels as a function of heart failure, previous heart attack, and PCI in the study group

		MPO [ng/ml] Average mean (SD)		
		All patients	DM	nDM
	Female Male	19.8 (8.0) 18.8 (8.0) *P =* 0.67	24.2 (6.3) 20.2 (9.9) *P =* 0.29	14.7 (6.9) 12.2 (4.8) *P =* 0.28
NYHA scale	0 ≥ 1	17.9 (6.6) 21.7 (10.4) *P =* 0.08	20.6 (7.1) 22.3 (12.5) *P =* 0.63	15.4 (5.0) 20.5 (5.4) *P =* 0.01
MI	No Yes	20.4 (10.0) 17.7 (4.9) *P =* 0.19	23.8 (10.4) 17.3 (5.3) *P =* 0.05	14.4 (5.8) 18.0 (4.7) *P =* 0.08
PCI	No Yes	19.2 (8.5) 19.0 (7.7) *P =* 0.88	22.5 (8.9) 20.5 (9.5) *P =* 0.56	15.3 (6.5) 12.3 (4.8) *P =* 0.36

*DM* Participants with diabetes type 2, *nDM* Participants without diabetes type 2, *MI* Myocardial infarction, *MPO* Myeloperoxidase, *NYHA* New York Heart Association, *PCI* Percutaneous coronary interventions, *SD* Standard deviation

Correlation analysis allowed to identify variables independently associated with the dependent variable (MPO) at the statistical significance level of *P* < 0.2. The multivariate analysis established type 2 diabetes and its correlation with elevated MPO levels. However, its effect on MPO levels was negligible (R^2^ = 0.08). Detailed results are shown in table 5.

**Table 5:** Correlation of T2DM and MPO levels

Dependent determinant MPO R = 0.29; R^2^ = 0.08; *P =* 0.02	beta	SD for beta	P
Independent determinant			
Diabetes type 2 (No/Yes)	0.25	0.12	0.05

MPO- myeloperoxidase, SD- standard deviation

### PON-1

There were no significant correlations between PON-1 and age, physical activity, dietary habits, substance use, anthropometric measurements, and physical exam results.

Also, no statistically significant correlations were discovered between PON-1 and the inflammatory indicator, or carbohydrate and lipid metabolism parameters, although negative correlations between PON-1 and glucose levels in the whole group (*P =* 0.05), as well as those between PON-1 and glycated hemoglobin in diabetic patients (*P =* 0.05), came close to statistical significance.

No correlation was observed between PON-1 levels and a history of heart attack in whole the group. The multivariate analysis likewise indicates a significant correlation between PON-1 levels and diabetes. The correlation, in this case, is negative (table 6).

**Table 6:** Correlation of T2DM and PON-1 levels

Dependent determinant PON-1 R = 0.43; R^2^ = 0.19; *P* < 0.01	beta	SD for beta	P
Independent determinant			
Diabetes type 2 (No/Yes)	−0.28	0.14	0.05

*PON1* Paraoxonase 1, *SD* Standard deviation

## Discussion

### Summary of main findings

In this study, we evaluated the association between type 2 diabetes mellitus and the concentrations of MPO and PON-1 in patients with IHD. Our results suggest that significant differences in enzyme concentration levels exist between patients with and without diabetes. Diabetes is associated with higher concentration of MPO and at the same time with lower concentration of PON-1. Differences in MPO and PON1 levels were maintained despite the statin treatment.

### Comparison with other studies

#### Impact of T2DM on MPO concentration levels in IHD patients.

The study revealed significantly higher MPO concentrations in the serum of diabetic patients. The results seem consistent with the data presented by Song et al., which reported higher plasma MPO levels in diabetic patients with IHD [19]. In the cited study the authors also observed a correlation between MPO levels and the progression of ischemic heart disease. The concentration of the enzyme was shown to be much higher in diabetic patients with advanced IHD and the disease progressed as a function of MPO levels. A stronger correlation was held for patients with higher HbA1c levels, what was not demonstrated in our study.

A study of Borato et al. showed a positive correlation between plasma MPO levels and classical inflammatory parameters and cardiovascular risk factors in diabetic patients [20]. Our study, however, did not reveal any significant correlations between MPO concentrations and the inflammatory indicator or selected parameters of carbohydrate and lipid metabolism. Interestingly, mean MPO levels were significantly higher in patients with heart failure, but only in the non-diabetic group.

A study published by Shao et al. showed that the levels of 3-chlorotyrosine and 3-nitrotyrosine, two typical products of MPO metabolism, tend to be elevated in HDL molecules isolated from patients with ischemic heart disease [21]. These products are likely to stand behind the oxidative damage to HDL particles. Our study showed a significantly higher concentration of MPO in patients with IHD and diabetes, which may be one of the mechanisms associated with HDL particles “dysfunction” in this patient group.

Zhang et al. demonstrated that in patients without T2DM, plasma glucose levels are positively correlated with MPO levels, which suggests that the latter may play an atherogenic role in patients with higher blood glucose [22]. Our study did not show such a correlation. The only parameter with an independent impact on MPO concentrations was the presence of diabetes alone. However, the actual impact of T2DM on MPO levels was minor, as evidenced by the low correlation coefficient in the model. In the other recently published observational study, it was shown that MPO levels were higher in obese compared with non-obese participants but did not differ between T2DM and control participants [23]. Our study did not reveal the correlation between MPO and patients’ BMI.

In the studies performed by Agarwal et al. and by Mahat et al. similar findings have been reported in patients with prediabetic state [24, 25]. The results of these studies showed statistically significant increase in the level of MPO in prediabetic subjects in comparison to control subjects.

A study by Heslop further confirmed that cardiovascular risk grows as a function of increasing MPO levels; the relationship is even stronger when hsCRP is considered [26]. It must be noted, however, that the study was conducted on patients who were not in treatment for T2DM .

Consistent with data from most recent studies, our results warrant the conclusion that plasma MPO levels, as an indicator of oxidative stress, may be useful for cardiovascular risk stratification in diabetic patient.

### Impact of T2DM on PON-1 concentration levels in IHD patients

In our study, significantly lower concentrations of PON-1 were observed in patients with diabetes than those without the disease. A decrease in PON-1 levels may be responsible for compromising the protective role of HDL particles in terms of inhibiting LDL oxidation. Another publication, which investigated PON-1 activity rather than its concentration, likewise reported a decrease in the activity of PON-1 in patients with diabetes, irrespective of the presence or absence of IHD [27].

Jamuna et al. observed a significant reduction in PON-1 activity, accompanied by a concomitant drop in HDL-C levels in diabetic patients as compared to healthy volunteers [28]. In addition, the study demonstrated that the progression of diabetes correlates with a further decrease in PON-1 activity. The correlation between low PON-1 levels in the serum and higher incidence and mortality rates of cardiovascular complications in patients with type 2 diabetes also was suggested [29]. Another recently published study suggests that PON-1 deficiency in T2DM is a gender-specific phenomenon, and that female patients are more affected than men [30]. The authors suggested that this could contribute to the partial loss of female cardiovascular advantage associated with T2DM. The results of our study did not reveal similar trend.

Even though most studies published thus far focus on enzyme activity rather than concentration, the overall trend seems to be the same. With that in mind, it has been proposed that the activity or concentration of PON-1 may serve as a better indicator of atherosclerotic risk in diabetic patients than the concentration of HDL-C alone. No clinical trials have unequivocally substantiated that claim thus a study performed by Jornayvaz et al. showed that myeloperoxidase was an independent, negative determinant of paraoxonase-1 activity [31]. The authors concluded that this may be one of the mechanisms by which it promotes HDL particles dysfunction and increases cardiovascular risk. The study published by Ferretti G. et al. demonstrated that the increase in oxidative stress in LDL and HDL of obese subjects is associated with a decrease in HDL-PON-1 activity [32]. The authors concluded that lower PON-1 activity could contribute to the greater risk of cardiovascular disease associated with obesity. This was confirmed by Cervellati C et al. [33]. However, in the cited above studies the activity of the enzymes was measured.

Another recently published study, which measured the levels of the enzymes in question, showed that plasma MPO concentrations show a significant inverse correlation with PON-1 levels in patients with stable and unstable angina [34].

Some researchers have observed that an imbalance between pro-oxidants and antioxidants may contribute to the progression of the instability of atherosclerotic plaques [35]. The results of our study suggest that a similar relationship should also be considered in patients with stable ischemic heart disease and type 2 diabetes, but the hypothesis requires additional studies on a larger patient group.

### The significance of study results

The results of our study suggest that T2DM is accompanied by elevated MPO levels. Since the latter may be associated with a greater cardiovascular risk in patients with type 2 diabetes, measuring MPO levels may aid risk stratification in this patient group. A significant reduction was also observed in the levels of PON-1, an enzyme with antioxidant and atheroprotective properties, in diabetic patients, which may further increase cardiovascular risk. As a result, the proportion of pro-oxidant and antioxidant enzymes becomes even more detrimental from the perspective of HDL-C function. All these factors increase the severity of the atherosclerotic process and may elevate cardiovascular risk despite normal HDL-C levels.

### Strengths and limitations of the study

A clear advantage of the study lies in its clinical nature and the assignment of patients to study groups, which ensured that the diabetic and non-diabetic groups would not differ significantly in terms of potential confounding variables. The only significant differences had to do with anti-diabetic drugs, which is the natural consequence of the disease, and the treatment’s potential impact on the study parameters should always be kept in mind.

Another strength of the study had to do with the advanced analytical methods used to perform measurements with a high degree of well-documented reliability. The analysis of correlations between the enzymes under study and several variables allowed to shed light on key clinical and lab relationships associated with T2DM.

The main limitation of the study was the small size of the study group. However, the inclusion criteria allowed to eliminate the most important potential confounding variables. Moreover, we did not include the duration of T2DM, however we have checked the HbA1c levels which indicate the degree of control of the diabetes. Also, due to the small size of the DM and nDM groups, multiple regression analysis models could only be constructed for the entire patient group, with diabetes introduced as an independent variable.

It must also be noted that the enzymes were measured at a single point in time and, for financial reasons, never repeated, which represents yet another weakness of the analysis. Also, it cannot be ruled out that the reason why no significant correlation was observed between parameters such as glucose or HbA1c and the levels of the studied enzymes was the limited size of the study groups.

Importantly, other studies into the correlations and the impact of enzymes on other parameters tend to rely on enzyme activity measurements. Our study focused on enzyme concentration levels rather than activity because of the methodological difficulties associated with measuring the latter. It should be kept in mind, however, that studies published thus far indicate a strong correlation between the two parameters [22]. Therefore, it seems probable that the significantly increased MPO concentration levels in diabetic patients in this study correspond to a greater activity of the enzyme in the group. In the future, the measurements of activity of MPO and PON-1 may be easier to implement in clinical practice. Lastly, we need to indicate that we did not check the polymorphisms in PON-1 and MPO, which may affect the expression of these enzymes.

## Conclusions

In patients with type 2 diabetes the profile of enzymes involved in HDL particles metabolism in patients with ischemic heart disease is significantly less favorable. It is associated with an increased concentration of myeloperoxidase, an enzyme with oxidative and atherogenic effects, and at the same time with reduced levels of paraoxonase-1, an enzyme with antioxidant and atheroprotective properties.

Our findings may have important prognostic significance; however, they need to be confirmed in a longer follow-up of a larger group of patients.

## Data Availability

The datasets used and/or analysed during the current study available from the corresponding author on reasonable request. Please contact the corresponding author: Dr. Katarzyna Nessler; Jagiellonian University Medical College, Department of Family Medicine; 31–061 Kraków, Bocheńska 4, Poland. Tel. + 48 12 430 55 93, Fax. + 48 12 430 55 84. katarzynanessler@gmail.com
